# Getting Digital Assets from Public–Private Partnership Research Projects through “The Valley of Death,” and Making Them Sustainable

**DOI:** 10.3389/fmed.2018.00065

**Published:** 2018-03-09

**Authors:** Wendy Aartsen, Paul Peeters, Scott Wagers, Bryn Williams-Jones

**Affiliations:** ^1^Hands4Grants, Leiden, Netherlands; ^2^BioSci Consulting, Maasmechelen, Belgium; ^3^Connected Discovery Ltd, London, United Kingdom

**Keywords:** precompetitive research, digital assets, innovative medicines initiative, translational research, infrastructure

## Abstract

Projects in public–private partnerships, such as the Innovative Medicines Initiative (IMI), produce data services and platforms (digital assets) to help support the use of medical research data and IT tools. Maintaining these assets beyond the funding period of a project can be a challenge. The reason for that is the need to develop a business model that integrates the perspectives of all different stakeholders involved in the project, and these digital assets might not necessarily be addressing a problem for which there is an addressable market of paying customers. In this manuscript, we review four IMI projects and the digital assets they produced as a means of illustrating the challenges in making digital assets sustainable and the lessons learned. To progress digital assets beyond proof-of-concept into widely adopted tools, there is a need for continuation of multi-stakeholder support tailored to these assets. This would be best done by implementing a structure similar to the accelerators that are in place to help transform startup businesses into growing and thriving businesses. The aim of this article is to highlight the risk of digital asset loss and to provoke discussion on the concept of developing an “accelerator” for digital assets from public–private partnership research projects to increase the chance that digital assets will be sustained and continue to add value long after a project has ended.

The Innovative Medicines Initiative (IMI) is the world’s largest biomedical research public private partnership. IMI projects are generating large amounts of medical research data. With the advent of big data approaches, any pretense that medical research is not a digital business has finally been exposed as the myth it has long been.

Accordingly, the IMI has funded numerous e-infrastructure and knowledge management projects (1). Each of these is targeted at the development of digital assets, enabling data services and platforms, which fill key gaps in the drug discovery value chain. Like any new product or service, enabling digital assets face a Valley of Death (2, 3). Often in a funded project, they are developed to proof-of-concept and a working prototype. But once project funding ends, the generated digital assets are at great risk of never being developed to the point where they achieve sustainable traction. The real risk is that the medical research data, generated by IMI projects and dependent on their co-developed digital assets, may become less accessible to the scientific community. It is, therefore, important to secure long-term sustainability and availability of project generated digital assets, as well as their integration within current ecosystems.

By its very nature, a public–private partnership includes different types of stakeholder who have varying perspectives and aspirations. The interest of pharmaceutical companies in projects that produce digital assets is mostly driven by the desire to enable research at a wide scale, which would be challenging for anyone company to achieve on their own. So, from perspective of the pharmaceutical industry, IMI projects are a means to work pre-competitively to address key gaps in the pharma value chain (4) [e.g., Drug Disease Model Resources consortium (DDMoRe),^1^ U-BIOPRED,^2^ European Translational Information and Knowledge Services (eTRIKS),^3^ and Open PHACTS^4^].

For smaller companies, with limited access to data specialists, public–private partnerships provide an opportunity to develop and access digital assets that are otherwise out of reach. For even smaller companies who are engaged in the development of data services and platforms, public–private partnerships provide the opportunity to participate in co-development. The assumption is that their core capabilities will be integral to the outputs of a project.

From the academic perspective, the interest lies in the production of innovative data services and platforms. The developed digital assets do have a potential impact on the ability of the wider scientific community to drive understanding of diseases (U-BIOPRED profiling data). Such an impact is also important to patient organizations, and regulators involved in these projects.

Regardless of the stakeholder, sustainability of the produced digital assets is essential for achieving the impact they are most interested in. However, achieving sustainability for digital assets is not straightforward. Our intent is to provoke a discussion to highlight the risk of losing digital assets after project funding ends and to propose a concept that will help to mitigate that risk.

## Challenges in Achieving Sustainability of Project Outcomes

There are no guidelines or financial regulations, no core agreement template or best-practice strategy, for sustaining a public–private partnership or its digital assets. It is not unlike a startup business. A startup is usually formed to solve a particular problem for which there is a large enough market of customers who will pay for that solution. At the end of the funding period of public–private partnerships, stakeholders have already paid for a solution, and there is usually no *a priori* consideration as to the size of the potential market.

### Startup Funding

A steady cash flow is needed to host, maintain, or even develop digital assets. Adequate and sustainable funding can be generated through offering services for a fee, sponsorship fees for membership in foundation or charity taking on the development of the digital assets, or both. Providing services requires an underlying organizational structure for which there will be costs to be covered. A particular challenge is that in order to obtain funding from other revenue sources such as a follow-on grant, there needs to be a legal structure, which requires funding to establish.

### Conflicting Business Models (Academia vs. Industry vs. Non-Profit)

To become financially viable, there has to be a clear understanding of the added value of the offered digital asset. Academic digital assets are often sustained by rolling components of tangible value into new projects such that these digital assets slowly evolve into core capabilities and community assets. In industry, there is a balance that has to be struck between leveraging digital assets from the public domain, integrating data and services with internal data and capabilities, and procuring proprietary services from commercial providers. This creates a large integration overhead. The non-profit business model is to make digital assets open and widely available. This is a promising solution, but even non-profits have to provide value in order to generate sufficient revenue to operate. While making access more open is necessary, this has to be achieved in a way that covers the cost of maintaining and developing that asset in the future. There is no such thing as a free lunch, and as digital assets become both increasingly important and vital to drug discovery, they also become increasingly complex and expensive to provide.

## Options for Sustaining Digital Assets

### Developing a Sustainable Business Model

Good business models are developed in a dynamic fashion as illustrated by a quote by Eric Ries from Lean Startup: “*Startups that succeed are those that manage to iterate enough times before running out of resources*” (5). Just providing a business model/strategy to a team of domain experts is only a start of a journey toward sustainability.

### Collaborative Business Model Generation

One of the biggest challenges in developing a sustainability model is communication. The academic mindset differs from the business mindset resulting in a communication gap. Sharing information, reaching consensus, open exchange of ideas and building upon each other’s work is part of the *modus operandi* in public–private partnership. To arrive at a sustainability model for digital assets this gap has to be bridged. It is also important to retain the engagement and commitment of the community which built the digital assets while simultaneously engaging other stakeholders. This is particularly the case with open source assets, and those which require engagement and adoption of the wider scientific community beyond those originally in an asset’s development.

The use of a business model canvas (6) or a derivative thereof to structure an iterative discussion is an effective approach for building a common understanding and integrating the perspectives of multiple stakeholders. In the eTRIKS project, an “asset maintenance canvas” was used to work with project partners to discuss and develop plans for sustainability, while DDMoRe and Open PHACTS used an approach more similar to a business model canvas. This is a very flexible approach, which allows a complete understanding of the sustainability requirements for any given asset that is understandable to all stakeholders and helps to facilitate the process.

### Sustainable Solutions

The role of providing further maintenance and development of digital assets developed in project can be carried forward by: (1) project partners, (2) an established commercial/nonprofit organizations, and/or (3) a newly formed external commercial/nonprofit organization. The biggest challenge lies in arranging for covering the costs of maintenance and further development. Depending on the nature of the digital assets, one option might be preferred over the other especially when considering intellectual property rights and obligations. Here, we present four different solutions, which illustrate real practical experience of sustaining digital assets, together with the inherent risks.

## Examples of Sustainability in IMI Projects

### U-BIOPRED

The project Unbiased BIOmarkers in the Prediction of REspiratory Disease (U-BIOPRED) was one of the first IMI projects to develop a plan to create the necessary infrastructure to support data integration, exploration, and preservation. Asthma is a diverse disease, and it is thought that many different phenotypes of asthma are not properly understood. U-BIOPRED created “handprints” that identify sub-phenotypes of asthma from large-scale phenotypic characterization of 1,025 patients. The effort involved the integration of multiple types of data including various high dimensional data types such as proteomics, metabolomics, genomics, and even a newly coined “breathomics.”

The size of the dataset, while large, was not large enough to consider it “big data.” It was, however, rich in terms of its complexity and the number of research questions it could address. In total, U-BIOPRED developed 12 analysis workflows, which were taken forward by separate teams. Over 100 publications have been planned. The complexity and richness of the dataset resulted in a number of challenges including managing the access for all the different parties, integration of different data types, and handling all the different analyses and teams that were engaged in analyzing the data. From the start, it was recognized that U-BIOPRED needed a platform to manage the data and knowledge that was being generated in the project.

During the early phase of the project, Janssen Pharmaceuticals, which was not an original partner, joined and brought their internal knowledge management (tranSMART) system into the project. The tranSMART platform allows for the parsing and exploration of translational research datasets curated into a common data model. Such a platform helps to solve the challenge of having multiple parties working on the same dataset as it provides a common repository that can be used to select out relevant subsets of the larger dataset. It is also being used to “explore” the dataset even to assist internal decision making within some of the U-BIOPRED pharmaceutical company partners. Such a use highlights the real value and the potential of a digital asset to improve the efficiency of research and new therapy development.

The U-BIOPRED collaboration is being maintained as part of the effort to develop a research agency within the European Respiratory Society (ERS) (7). “Data” is one of the main strategic activities of the ERS research agency and the support of the ERS has been essential to maintain the digital and other assets that were developed in U-BIOPRED. As was recently highlighted in the New England Journal of Medicine collaborations where nonprofits and patients have a central role are having a significant impact and may be the way to conduct translational research in the future (8). Nonprofits can act as a “backbone organization” (9) in translational research projects such as the Movember Foundations Global Action Plan (10). For U-BIOPRED, the ERS has been the “backbone organization” that is assuring that the maximal value is derived from the assets generated in the project. This could be a particularly important strategy for digital assets as non-profit organizations are able to take a long-term view and often see the value in making the most out of the assets we have and increasing the efficiency of medical research and development.

The inherent risk in this model is that it is dependent upon one organization and is a generalized model for sustaining all assets not just digital assets. Digital assets cannot be static like a biobank or even a dataset, they have to maintain a continued development trajectory if they are to remain relevant and gain traction.

### European Translational Information and Knowledge Services

The adoption of the tranSMART platform by U-BIOPRED stimulated further development of an IMI call topic focused on supporting IMI projects in their knowledge management needs—eTRIKS. As the project eTRIKS was being formed, Janssen Pharmaceuticals was working with the Pistoia Alliance to develop a nonprofit foundation to further develop tranSMART as open source software. The eTRIKS project was premised on using the tranSMART platform for supporting projects.

The initial remit for eTRIKS was to support 40 projects over the course of 5 years. At the end of 5 years, eTRIKS has supported 61 projects to varying degrees. A large part of that support has comprised substantial contributions to the tranSMART open source development effort. It has also included the development of a number of analytical tools through a collective effort termed eTRIKS Labs. As such, eTRIKS has produced a number of digital assets, which are in various stages of development.

The know-how developed over the course of the eTRIKS project will be sustained beyond the eTRIKS funding period as an eTRIKS Data Science Network that will be a consortium of providers that offer advice, support, development, curation across the data value chain. The digital assets will be maintained by the individual partners who had a major role in their development. eTRIKS has gone through a process of developing asset maintenance plans for each digital asset that include information on where it will be maintained as well as the provision of the necessary documentation.

The decision to sustain digital assets in a distributed network in eTRIKS was premised on two factors. Through the close relationship with the TranSMART Foundation, it was recognized that bundling the assets into a separate entity would create the risk that they may not be sustained if that entity did not achieve sustainable funding. Second, the lack of a stable structure or model that the assets could be transferred to, except for those that are being incorporated into the tranSMART platform. Another factor that played a role in this decision is that the individual partners contributed to the development of these assets and have a stake in seeing them sustained and gaining future value through further development.

There is, however, a risk with this model in that organizations with a primary education and research mission, are not focused on producing digital assets that attain the same degree of rigor as commercial products. Thus, these assets may never move through the digital asset “valley of death” to become more widely adopted or used beyond an academic research setting.

### Open PHACTS

The integration and application of diverse data for applied idea generation is a key step in early drug discovery research. Data from multiple sources both from the public domain and private sources are essential to understand the potential for new ideas, particularly relating to the relationship between potential drugs and targets. To lower the barriers to drug discovery, and power early precompetitive research, Open PHACTS built the first large-scale semantic drug discovery platform focused on answering key questions in early drug research (11). Through leveraging the experience of pharma companies, academics, and SMEs, Open PHACTS has been able to build a powerful open resource for drug discovery (12), which both simplifies data access and enables value-added research-focused workflows. Provisioning of fit-for-purpose research data services requires a stable, secure infrastructure, which has established the semantic data standards needed through close engagement of the wider academic community. Importantly, the Open PHACTS project recognized the need for digital assets sustainability early on, and has established the Open PHACTS Foundation (see text footnote 4) as a UK-registered non-profit charity, which maintains and develops the data services and infrastructure beyond the timeframe of the IMI project, which was completed in April 2016. The Open PHACTS Foundation is open to membership for any researcher or organization who share an interest in the applied use of research data for exploring biology, and who are willing to contribute to the running and maintenance of the Open PHACTS infrastructure and services.

Early in the process of establishing the Open PHACTS Foundation as the sustaining entity for the Open PHACTS IMI project output, several industrial partners (GSK, Janssen, Lilly, Roche, and Novartis) and academic partners (University of Vienna, University of Maastricht) joined the Foundation as partners. To ensure continued sustainability, a wide membership of private and public users is necessary to both maintain the current infrastructure and to develop and grow for the future. The Open PHACTS Foundation is a funded partner in H2020 projects, and a key pillar of sustainability remains leveraging the public–private opportunities to bring researchers together and better understand how to use data to better discover new drugs.

The risk in this model is having to fund the operating costs of the Foundation from sponsorships. There are any number of public/private partnerships that have come to the end of their funding period and look to subscribe to sponsors for further funding. This can lead to what has been informally expressed as “foundation fatigue.”

### Drug Disease Model Resources consortium

The Drug Disease Model Resources consortium set off to harmonize and standardize the way mathematical and statistical models are used and integrated in Pharmacometrics (PMX) to improve the quality, efficiency, and cost effectiveness of decision-making in Model-Informed Drug Discovery and Development (13). PMX model encoding is performed with a very heterogeneous set of software languages. Different notation and vocabulary as well as formats and file types are limiting the exchange of information. In order to make it easier to share and integrate models of compound, mechanism, and disease level data, DDMoRe faced the challenge of finding common ground to develop standards as a backbone for one platform to store and exchange models. DDMoRe succeeded in delivering a range of open-source exchange standards; a unified Model Description Language, the XML-based Pharmacometrics Markup Language a new exchange format for encoding of models, associated tasks, and their annotation (14), The Standard Output,^5^ a tool-independent standard exchange XML-based format for storing results. Systems like a repository to store models (Model Repository) and an Interoperability Framework as a tool to integrate software languages have been developed on top of the standards to demonstrate the possibility of sharing models and expertise within and between companies, regulatory authorities, and academic institutions.

Setting a standard is a collective piece of work, but a standard only becomes the standard if used by many. Additionally, further development and coordination of the standard requires industry and academic institutions to work together. The DDMoRe Foundation^6^ has been established as a Dutch-registered non-profit foundation to maintain and grow both standards and infrastructure for the benefit of the global community, pharmacometric experts, and the users of their model output alike. Early in the process of fundraising, two industrial partners (Servier and Pfizer) and two academic partners (Uppsala University and the University of Pavia) have joined the Foundation as partners. Recently, three more partners (GSK, Merck KGaA, and Leiden University) joined; however, to cross the “valley of death,” more partners need to be onboarded also representing SMEs and CROs. To assure that the digit assets become more widely adopted, ongoing negotiations with stakeholders like health authorities (e.g., EMA and FDA) have to materialize in concrete plans and actions for future alignment and development supporting the implementation of a sharing platform based on the DDMoRe standards.

Such a model carries the risk that the rate of adoption of standards does not achieve a large enough critical mass to provide sufficient funding for its continued development.

## Learning from the Startup Community

The challenge of having to sustain and further develop a digital asset after it has garnered some degree of proof-of-concept is not unlike the challenge faced by startups. Startups face what has been described as a “valley of death” where proof-of-concept has been achieved and early adopters are using the product. But, the process of gaining an increased market share and improving the product such that it can be scaled to meet a larger demand and satisfy customers is an enormous risk and requires funding. The startup community has addressed this at least in part by forming “incubators” or “accelerators” to help bring startups together, provide them some basic infrastructure and coaching, and provide a means for assessing which ones are most viable thereby reducing the risk to investors. These structures also help to reduce redundancy for standard activities such as contracts, accounting, and administrative staff.

A similar type of structure could be developed for the digital assets of IMI or other publicly funded digital assets. This could provide a common pool of knowledge about transforming digital assets into more sustainable products as well as provide a degree of flexibility in the type of support offered. Most importantly, it could provide a platform for those interested in the various digital assets that are being produced to assess which ones are worthy of further investment. It would also serve as a means of reducing the fragmentation that happens when assets are resolved back into the remit of the individual partners and help to protect the integrity of digital assets so that their progress can continue in a linear manner. There would be an additional advantage in that in such a structure one could find various digital assets that would be of use for a new project or even a new commercial project.

## Change in Paradigm

Through IMI, stakeholders have shown a clear commitment to drug discovery bottlenecks and enabling precompetitive research. For newly generated digital assets to become sustainable, they cannot be dependent on industrial funding alone. Similar to the model of the IMI, there needs to be continued public/private support to move these digital assets through the “valley of death” and support their continued development into more robust software and resources. This is not a unique challenge to IMI-generated digital assets, as digital science is becoming increasingly prevalent, this issue is relevant to the medical research community as a whole. For the most part, the funding streams for the translation of medical research are not the same as those which funded the research (13). Grant funding agencies are focused on research outputs such as new therapeutic targets and new technology. The risk is that there will be a lot of redundant efforts as new projects re-capitulate development and proof-of-concept efforts of preceding projects. There is too much to be done to waste resources in redundant efforts. IMI has lead the way in showing the potential of funding the development of tools and infrastructure that will enable the medical research community to capitalize on the potential of digital science. Now is the time for the whole community to embrace this change and deliver on that promise together in the future.

There are ongoing efforts to maintain digital assets. The IMI implemented a call topic on exploitation and sustainability of IMI project results where projects could place their assets into the call and different consortia could apply to have funding for maintaining those assets.^7^ The Pistoia Alliance is a nonprofit alliance that works on precompetitive projects to lower barriers to R&D innovation. They have successfully shepherded digital assets such as the tranSMART platform (15). ELIXIR is a European program that is focused on developing a European informatics infrastructure including bioinformatics.^8^ Grant funding agencies, industry, and other stakeholders such as disease foundations, regulators, and government bodies need to invest more into the refinement and maturation of digital assets. This would be best achieved by establishing digital asset accelerators that select the most promising digital assets and support their continued development under a common umbrella. This would bring the benefit of building know-how and experience, sharing best practices in making digital assets more robust and ultimately bringing them into a sustainable future. Experience in Silicon Valley is that accelerators do not need large amounts of funding to have substantial impact (16). Our hope is that this article can help to provoke a discussion that can lead to the support of more investment in sustaining the best digital assets so that the research outputs of projects remain accessible future projects do not have waste effort building digital assets anew.

## Author Contributions

WA, PP, SW, and BW-J contributed equally to this work.

## Conflict of Interest Statement

The author SW is the CEO of a company that provides consulting services to help manage projects and provide recommendations on sustainability solutions for projects and their digital assets. All other authors declare that the research was conducted in the absence of any commercial or financial relationships that could be construed as a potential conflict of interest.

## Footnotes

^1^http://www.ddmore.eu.

^2^http://www.europeanlung.org/projects-and-research/projects/u-biopred/home.

^3^https://www.etriks.org.

^4^http://www.openphactsfoundation.org.

^5^http://ddmore.eu/projects/so-standard-output.

^6^http://www.ddmore.foundation

^7^http://www.imi.europa.eu/sites/default/files/uploads/documents/apply-for-funding/open-calls/IMI2_Call11_CallText.pdf.

^8^https://www.elixir-europe.org/about-us accessed 16/01/2018
